# A revision of the genus *Osmoxylon* (Araliaceae) in Palau, including two new species

**DOI:** 10.3897/phytokeys.58.5292

**Published:** 2016-01-12

**Authors:** Craig M. Costion, Gregory M. Plunkett

**Affiliations:** 1 Botany Department, National Museum of Natural History, Smithsonian Institution, P.O. Box 37012, Washington DC 20013-7012; 2 Cullman Program for Molecular Systematics, New York Botanical Garden, 2900 Southern Blvd., Bronx, NY 10458-5126, USA

**Keywords:** Pacific Islands, taxonomy, Asteriids, Apiales

## Abstract

*Osmoxylon* Miq. (Araliaceae) is revised for Palau, Micronesia including descriptions of two new taxa *Osmoxylon leidichii* Costion, **sp. nov.** and *Osmoxylon ngardokense* Costion, **sp. nov**. Full descriptions are provided for all four Palau species, along with diagnostic field keys.

## Introduction

*Osmoxylon* Miq. (Araliaceae) is a genus of rainforest trees and shrubs from southeastern Asia and the western Pacific (Philipson 1979; Frodin and Govaerts 2003). All of the 60 currently recognized species are characterized by large umbelliform panicles composed of several to many three-branched inflorescence units. Each inflorescence unit terminates in a stalked head or umbellule that develops sterile baccate “pseudo-fruits” and two lateral stalked heads or umbellules with fertile bisexual flowers and fruits. Other characteristic features of the genus include ligule-shaped stipules that clasp the stem and conspicuous rings or crests that circle or spiral around the petiole base. Like most araliads, the calyx is inconspicuous, forming a highly reduced rim around the inferior ovary, but the genus is unusual in the family in that all species have fused or united petals, forming a distinctively tubular corolla. The genus has not received a modern comprehensive revision, but several regional treatments of *Osmoxylon* have made important contributions to its taxonomy from Malesia (Philipson 1979; Frodin 1998), New Guinea (Philipson 1995), and the Solomon Islands (Conn and Frodin 1995), and included the transfer of *Boerlagiodendron* Harms to *Osmoxylon* (see Philipson 1976; Frodin 1998). The highest species diversity for the genus occurs in the Philippines (17–19 spp.). Its range extends north to Taiwan (1), east into the Caroline and Marianna Islands (4) and south to the Wallacea region of Indonesia (11). From Wallacea its distribution extends west only to Borneo (2) but eastward across New Guinea (11) and the Solomon Islands (14) to Vanuatu (1).

Three species are currently recognized in Palau: *Osmoxylon oliveri* Fosberg & Sachet, *Osmoxylon pachyphyllum* (Kaneh.) Fosbeg & Sachet, and *Osmoxylon truncatum* (Kaneh.) Fosberg & Sachet. The first record of the genus in the archipelago seems to date from the Japanese era, with a collection made in 1929, as recorded by Kanehira (1931) and identified as *Boerlagiodendron pulcherrimum* (Vid.) Harms. This species was originally described in *Osmoxylon* by Fernández-Villar (1880) based on material from the Philippines, but later transferred to Harms’ (1894-1897) segregate genus. Shortly thereafter, Kanehira (1934) described two additional species, *Boerlagiodendron truncatum* Kaneh. and *Boerlagiodendron pachyphyllum* Kaneh. While Palau was under the administration of the United States, all three species were transferred to *Osmoxylon* by Fosberg and Sachet (1980), following Philipson’s (1976, 1979) treatment of the genus in Malesia. In the same publication, Fosberg and Sachet segregated the Palauan material assigned to *Boerlagiodendron pulcherrimum* (otherwise endemic to the Philippines) as a distinct species, *Osmoxylon oliveri*.

All three currently recognized species are known to occur on Palau’s largest island, Babeldaob. *Osmoxylon truncatum* is only known from Babeldaob, but both *Osmoxylon oliveri* and *Osmoxylon pachyphyllum* have hitherto also been recorded from Palau’s limestone islands. Recent collections, however, provide evidence that specimens identified as *Osmoxylon pachyphyllum* from Palau’s aforementioned islands represent a distinct, undescribed species and that the original concept of *Osmoxylon truncatum* requires revision. Furthermore, another new species of *Osmoxylon* was recently discovered while establishing a forest-dynamics plot on Babeldaob. Herein, we here describe these two new species and combine the two previously known species *Osmoxylon oliveri* and *Osmoxylon truncatum*. A dichotomous key to the four known members of the genus in Palau is provided with a list of diagnostic characters for each species so they may be more easily distinguished by non-experts. These taxa tend to be encountered infertile in the field, resulting in great confusion in their identification, and we therefore provide two separate diagnostic keys, one based solely on vegetative material and a second for fertile collections.

### Key to vegetative material of the Palau species of *Osmoxylon*

**Table:** 

1	Leaf lobes 5–9	**2**
2	Leaf lobes 5–7; stipules glabrous, strongly folded or recurved with tip sharp to the touch; teeth exserted or protruding from margin	***Osmoxylon pachyphyllum***
2’	Leaf lobes 7–9; stipules with tannish pubescence, clasping stem, flattened; teeth inserted, each tooth located within a crenulation in the margin	***Osmoxylon leidichii***
1’	Leaf lobes 9–15	**3**
3	Leaf lobes 9–11; stipules entire, glabrous; each prominent secondary vein branched, terminating in 1 or 2 serrations; junction of midrib and secondary veins nearly perpendicular, secondary veins then curving to a 30–45°angle	***Osmoxylon nardokense***
3’	Leaf lobes 11–15; stipules with distinct teeth or ciliate crests, more or less glabrous; prominent secondary veins branched, terminating in 2 or 3 serrations; junction of midrib and secondary veins at 30–45°angle	***Osmoxylon truncatum***

### Key to fertile material of the Palau species of *Osmoxylon*

**Table:** 

1	Inflorescence < 20 cm diameter, < 25 flowers or fruits per fertile umbellule	**2**
2	Fertile umbellules 20–30 per inflorescence, borne on peduncles that are not distinctly jointed; fertile fruits globose, c. 20 per umbellule	***Osmoxylon leidichii***
2’	Fertile umbellules 12–15 per inflorescence, borne on distinctly jointed peduncles, fertile fruits oblong in outline, angled and flattened, 3-5 per umbellule	***Osmoxylon pachyphyllum***
1’	Inflorescence c. 20–30 cm in diameter, > 25 flowers or fruits per fertile head/umbellule	**3**
3	Flowers 20–40 per umbellule, borne on minute pedicels; fertile fruits distinctly globose, shiny dark purplish-black	***Osmoxylon ngardokense***
3’	Flowers 45-80 per head, sessile at anthesis, borne on a cone-shaped receptacle, pedicels form as fruits mature; fertile fruits obpyramidal (corn-kernel shaped), white-green maturing to dull purple from apex toward the base	***Osmoxylon truncatum***

## Taxonomic treatment

### Osmoxylon leidichii

Taxon classificationPlantaeApialesAraliaceae

Costion sp. nov.

urn:lsid:ipni.org:names:77151879-1

Fig. 1

Boerlagiodendron pachyphyllum Kaneh. p.p., Bot. Mag. Tokyo, 48: 401, 1934
Osmoxylon leidichii Syntype: Palau. Aimeliik: 1933, R. Kanehira 2452

#### Type.

Palau. Koror: Ngeremdiu Beach, 07°15'20.22"N, 134°26'37.98"E, 18m, 6 Jun 2014 (fr), C. Costion 3711 (holotype: NY; isotype: US, BNM).

#### Description.

Small to medium sized tree, 10–12 m tall, branched. Leaf blades palmately lobed, up to 45 cm long and wide, glabrous, with 7–9 rhombic lobes; margin with serrations minute and distinctly inserted or in tiny indentations of the blade, barely exceeding the margin itself, only one per secondary vein; prominent secondary veins 7 or 8 per lobe, meeting the mid-rib at a 30–45°angle; petioles up to 50 cm in length; petiolar crests 1–4, circular, re-curved, entire, with tannish pubescence along margins; stipule flattened and appressed to the stem, broadly attenuate to the apex, fleshy (not stiff), with brown flakey or papery margins and brownish pubescence, tip soft to touch, not firm. Inflorescence 10–15 cm in diameter, primary axis bearing 20–30 secondary inflorescence units, secondary axis (from primary axis to the point where the lateral fertile umbellules are attached) c. 2 cm long, with c. 30 pinkish to crimson, baccate pseudo-fruits, each 2 mm long and 2–3 mm in diameter; peduncles c. 3 cm long, light green, not distinctly jointed. Fertile flowers 10–20 per umbellule, each with a yellowish, fused or united, cup-shaped calyx, c. 2 mm long; corolla tube bright orange, 5 or 6 lobed, 3 mm long; stamens alternate to the petal lobes, strongly exserted; ovary inferior, whitish-green, stigmas sessile. Fruits 10–20 per umbellule, each with 5 or 6 locules (each 1-seeded), globose-ovoid, 7–8 mm long, 6–7 mm in diameter, turning white (when immature) then maturing to pale pink, tightly clustered at maturity, forming a distinct hemispheric or mound-shaped infructescence.

**Figure 1.**
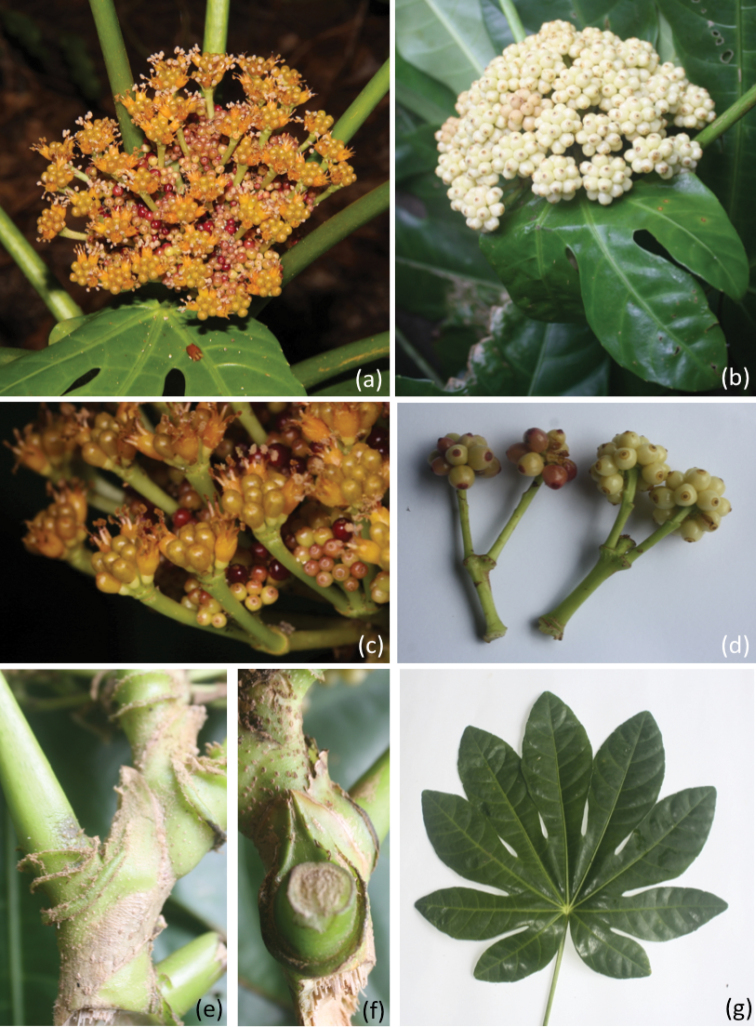
*Osmoxylon leidechii* **a** compound inflorescence with flowers **b** compound inflorescence with fruits **c** fertile flowers and sterile fruits **d** individual inflorescences with fruits **e** petiolar stipule side view and stipule crests **f** petiolar stipule overhead view **g** leaf.

#### Notes.

*Osmoxylon leidichii* occurs across the limestone islands of Palau on karst and coral substrates and within this range it appears to be common. It has been previously confused with *Osmoxylon pachyphyllum*, but is distinguished by its 7–9 rhombic leaf lobes, its appressed, flakey stipules, and its inflorescences, which bear many more fruits that are each smaller and more globose. One of the syntypes (*R. Kanehira 2452*) cited in the protologue of *Boerlagiodendron pachyphyllum* belongs to this new species. We are happy to name this species after the Palau resident and naturalist, Ron Leidich, whose generosity enabled the discovery of this species and for his inspirational knowledge and enthusiasm about Palau’s natural history.

#### Specimens examined.

**Palau.** Koror State: Ngeremdiu Beach, 6 Jun 2014 (ster.), C. Costion 3708 (US); Ngeremdiu Beach, 6 Jun 2014 (fl.), C. Costion 3709 (BNM); Ngeremdiu Beach, 6 Jun 2014 (fl.), C. Costion 3710 (NY, US); Ngeruktabel Island, 26 Jun 1982 (fr.) Hobdy 1547 (BISH); uninhabited coral island, 14 Aug 1933 (ster.) R. Kanehira 2452 (TI, FU); Ngeruktabel Island, 8 Aug 2007 (fr.) M. Balick 4511 (BNM, NY); Ngeruktabel Island, along path from boat landing to German Lighthouse, 07°15'50.3"N; 134°26'45.9"E, 135 m, 9 Nov 2013 (ster.), G.M. Plunkett 2707 (BNM).

### Osmoxylon ngardokense

Taxon classificationPlantaeApialesAraliaceae

Costion sp. nov.

urn:lsid:ipni.org:names:77151880-1

Fig. 2

#### Type.

**Palau.** Melekeok: Ngardok forest dynamics plot, 07°30'36.97"N, 134°36'28.04"E, 50 m, 17 Jul 2014 (fr.) C. Costion 3721 (holotype: NY; isotype: BNM, US).

#### Description.

Small understory tree, 7–10 m tall, unbranched. Leaf blades large with distinct celery smell when crushed, up to 60 cm long and 75 cm wide, with 9–11 lobes; margin weakly serrated, serrations exserted or protruding from margin and spaced far apart, generally 1 per secondary vein or up to one between secondary veins; prominent secondary veins 8–13 per lobe, meeting the mid-rib at a near 90°(perpendicular) angle then curving to a 30–45°angle; petioles up to 92 cm in length; petiolar crests 3, circular, with papery edge and minutely toothed; stipule 3.5–4 cm long, deeply furrowed, slightly appressed to stem and mildly recurved on the axial side, tip not sharp or firm. Inflorescence 20–25 cm in diameter, primary axis bearing 30–40 secondary inflorescence units, secondary axis (from primary axis to where lateral umbellules are attached) c. 3.5 cm long, sterile fruits not seen; lateral peduncles jointed, c. 4–5 cm long, bottom segment 1.2–1.5 cm, top segment 2.5 cm; dark purplish-black in color. Fertile flowers 20–30 per umbellule, with light-green, fused or united, globose to cup-shaped calyx, 2–2.5 mm long; corolla tube bright yellow-orange, 4–5 lobed, 3 mm long, 1.5–2mm wide; stamens alternate to the petal lobes, partially exserted; ovary inferior, greenish, stigmas sessile. Fruits 30–40 per umbellule, with 5 locules (each 1-seeded), globose, 3–6 mm in diameter, turning dark blackish-crimson; fruiting umbellules globose, 2–2.5 cm in diameter at maturity, spaced apart, not densely packed.

**Figure 2.**
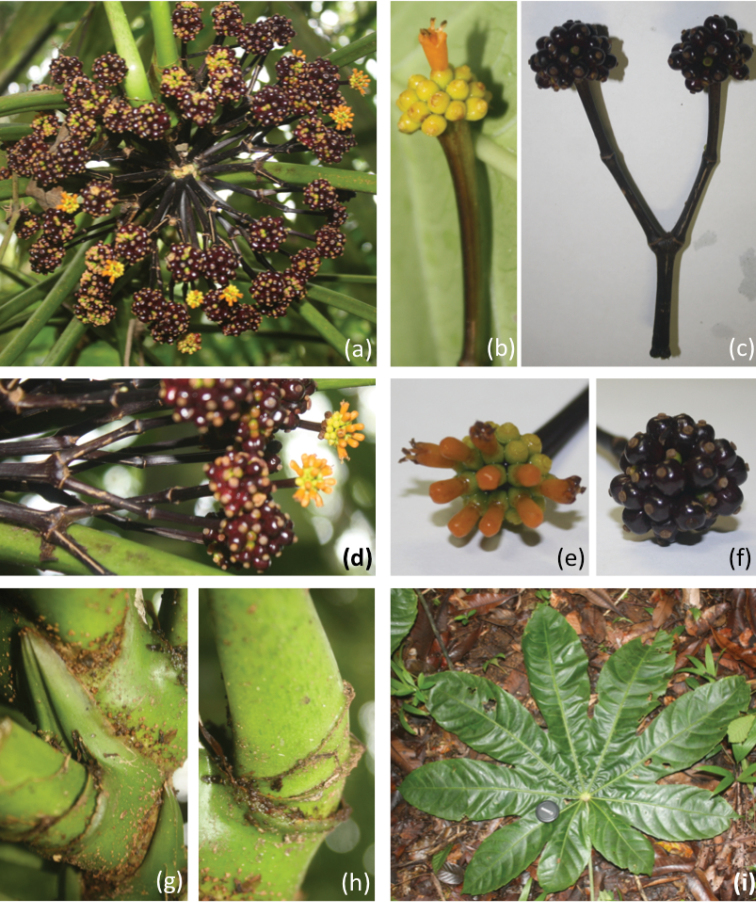
*Osmoxylon ngardokense* **a** compound inflorescence **b** inflorescence **c** inflorescence with mature fruits **d** inflorescences fruiting and flowering **e** flower head up close **f** mature fruits up close **g** petiolar stipule **h** petiole crests **i** leaf.

#### Notes.

*Osmoxylon ngardokense* is so far known only from the type locality, with volcanic soil, near Lake Ngardok on Babeldaob, within the Ngardok Nature Reserve, for which the species is named. This species is clearly distinct from the other Palau taxa of *Osmoxylon* by its large 9–11 lobed leaves, large compound inflorescence with inflorescences widely spaced, and dark crimson globose fruits in globose clusters of 30–40.

#### Specimens examined.

**Palau.** Melekeok State: Ngardok Nature Reserve in Ngardok forest dynamics plot, 21 Jul 2014, 23 Jul 2014, C. Costion 3895 (BNM), C. Costion 3725 (NY).

### Osmoxylon truncatum

Taxon classificationPlantaeApialesAraliaceae

(Kaneh.) Fosberg & Sachet

Fig. 3

Boerlagiodendron truncatum Kaneh., Bot. Mag. Tokyo, 48: 403, fig. 2, 1934.
Osmoxylon oliveri Fosberg & Sachet, Smithsonian Contr. Bot. 45: 16. 1980.
Osmoxylon truncatum Type. Palau. Ngardmau: near Dudui’s homestead, 2 Apr 1966 (fr.,fl.), Cheatham 54 (holotype: US!; isotype: NY!, BISH!).

#### Type.

Palau. Aimeliik State: 2 Aug 1933, R. Kanehira 2364 (holotype FU!; isotype: NY!).

#### Description.

Small to medium-sized understory tree, 10–20 m tall, branched. Leaf blades palmately lobed and large, up to 80 cm long and 85 cm wide, glabrous, generally with 11–15 lobes, strongly serrated; serrations protruding from margin, 2 or 3 in between each prominent secondary vein; prominent secondary veins meeting the mid-rib at a sharp 45°angle; petioles up to 1.2 meters long, petiolar crests 3 or 4, circular, ciliate; stipule appressed to stem, shallowly furrowed on top with 1–3 ciliate crests resembling horizontal lines of teeth or wart-like projections; margin of stipule papery and tannish, expanding in towards the center as the stipule matures. Compound inflorescences 20–30 cm in diameter, primary axis bearing 20–40 secondary inflorescence units, secondary axis (from primary axis to where lateral umbels are attached) 3–6 cm long, supporting an umbel of 20–30 dark purple to blackish baccate pseudo-fruits up to 1 cm in diameter; peduncles jointed, purple, 5–7 cm long, with bottom segment 2–6 times shorter but becoming longer with maturity. Flowers 45–80 per head with yellow, angular, bright yellow calyx crowning the ovary; corolla tube bright reddish-orange, 5-lobed, 4 mm long; stamens alternate to the petal lobes, strongly exserted; ovary inferior with sessile stigma. Fruits pedicelate, obpyramidal, resembling corn kernels, each c. 1 cm long, 1 cm wide, greenish to white, maturing with a dull purple apex and striations down to the base; fruiting umbellules c. 5 cm long, 4 cm wide, mulberry shaped, with up to 80 fruits densely pressed together.

**Figure 3.**
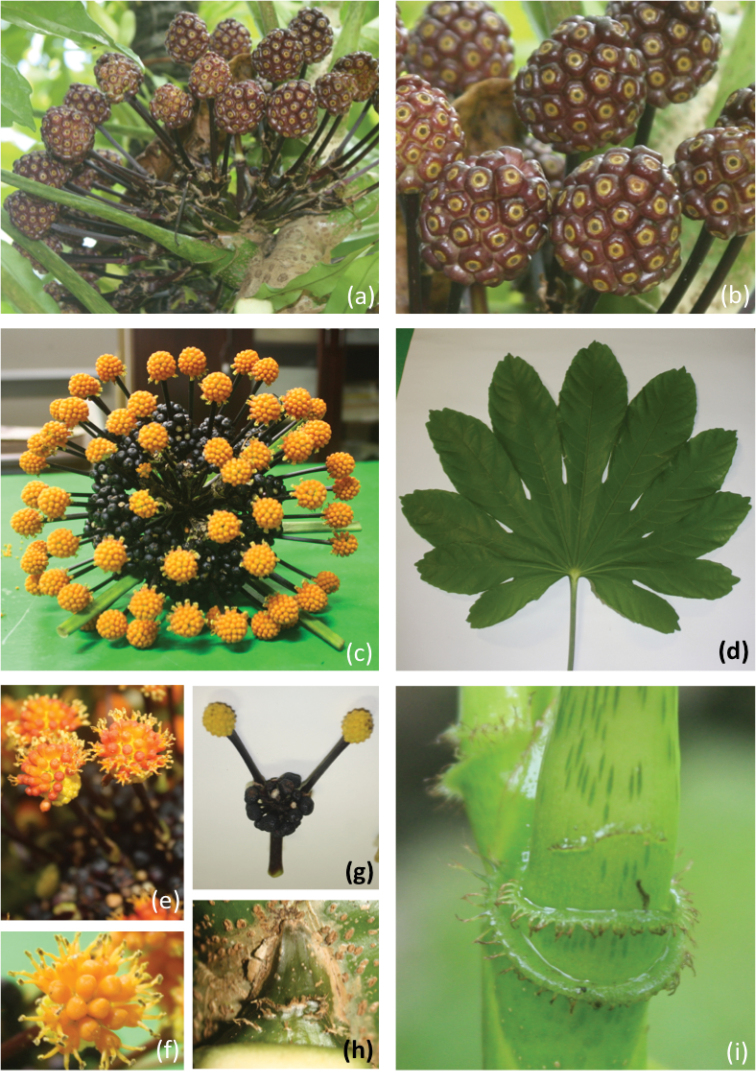
*Osmoxylon truncatum* **a** compound infructescence with mature fruits **b** mature fruits **c** compound inflorescence with un-opened flowers **d** mature leaf **e** one mature inflorescence **f** flower head **g** one fertilized inflorescence, without corollas **h** petiolar stipule **i** petiole crests.

#### Notes.

*Osmoxylon truncatum* is common in both the limestone and volcanic islands of Palau and is often found in villages near dwellings. The flowers are used for decorations in traditional and modern customs and events. The species is distinguished from the other Palau *Osmoxylon* taxa by its leaves with 11–15 lobes, ciliate crested stipules, and its much larger inflorescences with up to 80 flowers and fruits.

#### Specimens examined.

**Palau.** Aimeliik State: along road to power plant, Dec 2014, Costion 3987–3989, (BNM, US); Airai State: just south of main entrance to airport, 07°21'49.0"N; 134°31'54.1"E, 64 m,12 Nov 2013 (fl., fr.), G.M. Plunkett 2716 (BNM, NY); Ngetkib, agroforest, 7 Aug 2007 (fr.), M. Balick 4475 (BNM, NY); near airport, 15 Oct 1978 (fl.) Shearard & Spence 89 (BISH); Babeldaob, 1 Nov 1933 (fl.) Herre 71 (BISH); Kaiguru, 15 Apr 1936 (fl.), Takamatsu 1611 (BISH); Koror State: Koror, BNM botanical garden, 9 Mar 2007 (ster.), Kitalong 30907 (BNM); Coral island, Aug 1932, Kanehira 1853 (FU); Aug 1929, Kanehira 129 (FU); BNM botanical garden, 5 Dec 2014, Costion 3980–3986, 3990–4000 (BNM, US); Melekeok State: Aug 1932, Kanehira 2057 (FU); Ngaraard State: tributary of Ngereakl R., 20 Jan 1978 (fr.) J. Canfield 397 (BNM, BISH); Ngarchelong State: west of Pkulrengerelong, 3 Jan 1978 (ster.), J. Canfield 304 (BMM); Ngaremlengui State: upper Ngarmiskan R., 8 Dec 1978 (fr.) J. Canfield 650, 651, (BNM); Ngatpang State: Mechutelngatpang, 5 Aug 2008 (fr.), M. Balick 4594 (BNM, NY); Ngiwal State: along Ngareboku R., 17 Jan 1978 (ster.), J. Canfield 362 (BNM); Aug 1932, Kanehira 2065 (FU), Aug 1932, Kanehira 2066 (FU).

### Osmoxylon pachyphyllum

Taxon classificationPlantaeApialesAraliaceae

(Kaneh.) Fosberg & Sachet

Fig. 4

Boerlagiodendron pachyphyllum Kaneh., Bot. Mag. Tokyo, 48: 401, 1934.

#### Type.

Palau. Aimeliik: 1933 (fr.), R. Kanehira 2301 (lectotype: FU!, here designated; isolectotype: TI!).

#### Description.

Small to medium sized understory, tree 7–15 m tall, sparsely branched. Leaves palmately lobed, variable in size, up to 60 cm long and 65 cm wide (generally smaller), with 5–7 lobes; margins sparsely dentate with serrations exserted from margin, 1 per prominent secondary vein or alternating between veins; prominent secondary veins meeting the mid-rib (near) perpendicular then curving to a 30–45°angle; petiolar crests 1–2, rarely 3, firm with sharp edges, sparsely ciliate; stipules not appressed to stem, strongly recurved, glabrous, tip sharp to the touch. Inflorescence 7–15 cm in diameter, primary axis bearing 12–15 secondary inflorescence units, secondary axis (from primary axis to where lateral umbellules are attached) c. 2.8 cm long, with c. 30 pinkish-red baccate pseudo-fruits, c. 4 mm in diameter; peduncles jointed, c. 2.6 cm long, top segment shorter, maturing to equal the length of the bottom segment, green. Fertile flowers 10–15, with greenish-yellow, fused calyx crowning the ovary; corolla tube yellowish-orange, 6 lobed, c. 5 mm long; stamens alternate to the petal lobes, strongly exserted; ovary inferior, stigmas sessile. Fruits flat sided, 1.2–1.5 cm long, 0.7–0.8 cm wide, greenish with reddish-dull purple apex and striations down to the base, c. 3–5 per umbellule; fertile fruiting umbellules loosely organized with distinct peduncles, 1.5–3 cm diameter.

**Figure 4.**
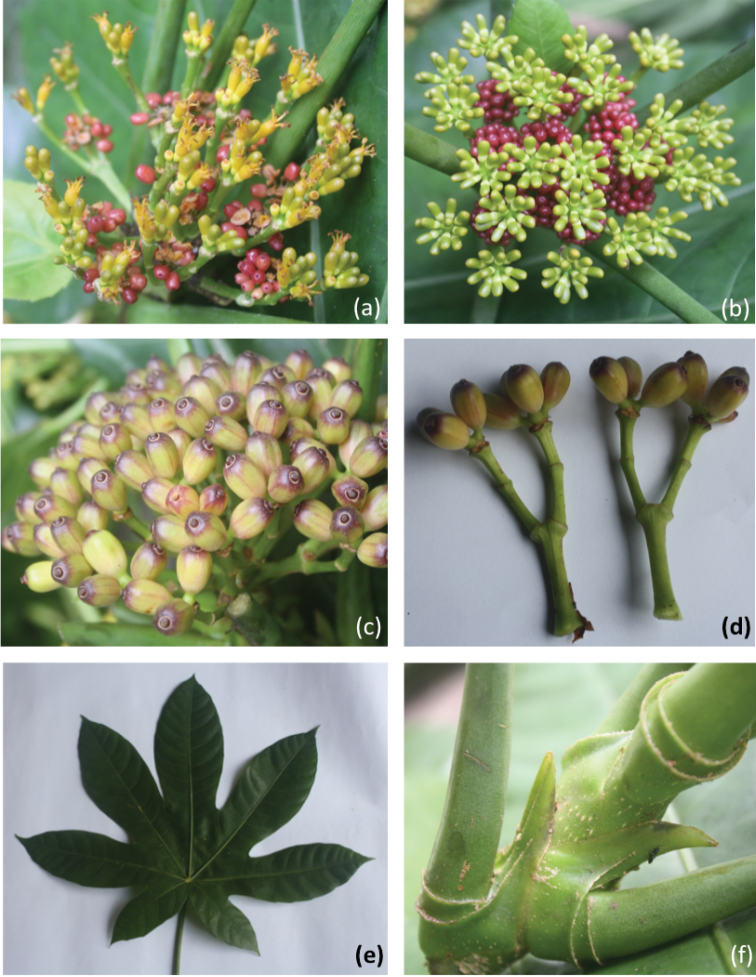
*Osmoxylong pachyphyllum* **a** compound inflorescence with mature flowers **b** compound inflorescence with immature flowers **c** compound inflorescence with mature fruits **d** two inflorescences with mature fruits **e** mature leaf **f** petiolar stipule and petiole crests.

#### Notes.

As circumscribed herein, *Osmoxylon pachyphyllum* is known only from volcanic soils on Babeldaob Island. Previous collections of this species from the limestone islands (including a syntype, R. Kanehira 2452) are now referred to the new species *Osmoxylon leidichii*. In addition to its geography and ecology, *Osmoxylon pachyphyllum* can easily be distinguished by its 5–7 lobed and weakly serrated leaves, its oblong, large, angled fruits, and its umbellules, which have very few (3–5) fruits, compared to all other species known from Palau. The stipule at the petiole base is also distinctive among the Palauan members of the genus in being strongly recurved, pointing away from the stem, and with a noticeably sharp tip.

#### Specimens examined.

**Palau.** Aimeliik State: slope of Ngetchum, 28 Dec 2005 (fl.) C. Costion 894 (BNM); Jul 1933, Kanehira 2301 (FU); 30 Jul 1933, Kanehira 2311 (FU); Babeldaob Island, south central Babeldaob, SW of Mt. Yekigoroto, 2 Sep 1965 (fr.), R. Fosberg 47677 (BISH); Babeldaob Island, 17 Apr 1938 (flw), Hatusima 5021 (FU); Babeldaob Island, 18 April 1938, Hatusima 5053 (FU); Melekeok State: Ngardok Nature Reserve in Ngardok forest dynamics plot, Jul 2014 (fr.,fl.) C. Costion 3779, 3780, 3781, 3802 (BNM, US); Ngardmau State: 2005 (fr.), C. Costion 90 (BNM); Ngertebechel watershed south of waterfall, 15 Jul 2005 (fl.), C. Costion 449 (BNM); Ngaremlengui State: along trail from Mr. Ha’s quarry to Parkia population and waterfall, 07°32'39.6"N; 134°34'07.2"E, 131 m, 5 Nov 2013 (bud, fl., fr.), G.M. Plunkett 2686 (BNM, NY); Ngechesar State: along Iliud ra mesiual historic trail, 99 m, 7 Jun 2014 (fr.) C. Costion 3712 (BNM, US), 7 Jun 2014 (fr.) C. Costion 3713 (NY), 7 Jun 2014 (fl.) C. Costion 3714 (NY), 7 Jun 2014 (fr.) C. Costion 3715 (BNM, NY, US), 7 Jun 2014 (fl.) C. Costion 3716 (BNM, US), 7 Jun 2014 (fl.) C. Costion 3717 (US), 7 Jun 2014 (fr.) C. Costion 3719 (NY, US)

## Discussion

### Vegetative characters

The most useful characters for distinguishing among the species of *Osmoxylon* present in Palau are summarized in Table 1. Of these, leaf size can vary considerably between individual trees within each species. The number of lobes sometimes varies due to slower development of basal lobes. The presence of distinctive veins present at the leaf base often indicates an undeveloped lobe. We found that the number of lobes can be useful in the field for distinguishing among species if caution is used in inspecting several leaves per tree. Optimally, this information should be recorded as label data for herbarium specimens, but because most specimens do not include such data, leaf-lobe number alone is not sufficient for identifying herbarium specimens, especially given the tendency of collectors to select smaller leaves (often the reduced ones emerging directly under inflorescences) that are easier to press. The leaf-margin serrations can also be useful, particularly the number of teeth per prominent secondary vein. In this regard, *Osmoxylon leidichii* is very distinct from the three other Palauan species in having teeth occurring in indentations in the margin with the tooth apex not exceeding the margin. The angle of the junction between the secondary veins and primary veins cannot be used alone, but can help rule out two out of the four Palau species, and the stipule also has diagnostic value. The petiolar crests or rings are sometimes used to distinguish *Osmoxylon* species in other geographical regions. Among the Palauan taxa, the number of petiolar crests can vary within each species, but they have somewhat different margins (e.g., ciliate or nearly entire). These features are sometimes absent or not persistent and are therefore not sufficiently distinct to distinguish the Palau species in the absence of other characters.

**Table 1.** Diagnostic morphological characters useful for distinguishing the Palau *Osmoxylon* species.

Diagnostic Characters	*Osmoxylon leidichii*	*Osmoxylon ngardokense*	*Osmoxylon truncatum*	*Osmoxylon pachyphyllum*
LEAVES				
Mature leaf lobes	7–9	9–11	11–15	5–7
Serrations	Inserted, 1 per secondary vein	Exserted, 1 or 2 per secondary vein	Exserted, 2 or 3 per secondary vein	Exserted, 0 or 1 per secondary vein, sometimes absent
Secondary vein orientation	30–45°angle with midrib	Near 90°angle with midrib	30–45°angle with midrib	Near 90°angle with midrib
Stipule	Appressed to stem, tannish pubescence	Appressed to semi-recurved, glabrous	Appressed to stem, ciliate crests/teeth	Recurved, glabrous, tip sharp
FLOWERS				
Compound umbel dia.	10–15 cm	20–25 cm	20–30 cm	7–15 cm
No. inflor-escences per compound umbel	20–30	30–40	20–40	12–15
No. flowers per head	10–25	20–40	45–80	10–15
Fertile peduncles	c. 3 cm, not distinctly jointed	4–5 cm, jointed, bottom segment shorter maturing to equal top segment	5–7 cm, jointed, bottom segment 2–6 times shorter	c. 2.6 cm, jointed, top segment shorter maturing to equal bottom segment
FRUITS				
No. per head	10–25	20–40	Up to 80	3–5
Head shape	Globose	Globose	Oblong	Umbel
Head size	1.5–2 cm dia.	2–2.5 cm dia.	4–5 cm dia.	1.5–3cm dia.
Fruit size	0.7–0.8 × 0.6–0.7 cm	0.3–0.6 cm	1.0 × 1.0 cm	1.2–1.5 × 0.8 cm
Shape	Globose-ovoid slightly angled	Distinctly globose	obpyramidal (corn kernel)	Oblong, flattened sides
Color	White maturing to pinkish	Shiny dark purple-blackish	White-green maturing to dull purple from apex	Yellow-green with maroon-dull purple striations and apex
Compound inflorescence shape at fruit maturity	Hemispheric	Globose	Globose	Umbel-shaped

### Reproductive characters

Diagnostic reproductive characters include the size of the inflorescence, the number of secondary inflorescence units, the number of flowers per head or umbellule, and various features of the peduncles of the fertile heads. The fertile heads or umbellules of three out of the four Palau species have distinctly jointed peduncles, where caducous bracts are present. The peduncles also differ in the proportional lengths of the upper or lower segments (above and below the bracts or bract scars). Bracts tend to subtend each segment of the compound inflorescence but are rarely persistent and thus their morphology does not provide reliable characters. Floral characters are similar among the four species, each having yellow-orange corollas with 4-6 lobes and cup-shaped or globose calyces surrounding the ovary. When present, features of the mature fertile fruits (their size, shape, and color, as well as the number of fruits per umbellule) can be used to distinguish unambiguously among all four Palau species and seem to be the most reliable diagnostic characters. This suggests that fruiting material is particularly important for understanding species limits within the genus and is particularly desirable for recognizing and describing new entities.

### Resolving the identity of *Osmoxylon truncatum*

*Osmoxylon truncatum* was previously known only from two collections, neither of which contained mature fertile parts (type: R. Kanehira 2364, and R. Kanehira 2303). Thus, resolving its correct identity required considerable effort. All other records attributed to this species were misidentified collections of either *Osmoxylon oliveri* or *Osmoxylon pachyphyllum*. The type specimen contains only immature flowers and no fruits are known. Kanehira (1934) noted that this species differs from the other Palauan taxa in having central infertile umbellules borne on peduncles that are longer than those of the lateral, fertile umbellules, and that the inflorescence heads are smaller. These characters, however, are consistent with the immature state of the inflorescence found on the type of *Osmoxylon oliveri*. Indeed, smaller inflorescences of *Osmoxylon oliveri* tend to occur in the deeply shaded understory of forests, whereas trees growing in open habitats tend to have larger inflorescences. Kanehira (1934) also distinguished *Osmoxylon truncatum* based on its having leaves with a truncate base and 7 lobes. However, truncate leaf bases have also been observed in some specimens of both *Osmoxylon oliveri* and *Osmoxylon pachyphyllum*. The number of lobes can be a useful guide for identifying species of *Osmoxylon* in Palau, but is not suitable as a primary feature for delimiting new species without mature flowers and/or fruits, as was done in the case of *Osmoxylon truncatum*. Young leaves and those directly subtending inflorescences have fewer lobes than the leaves at full maturity, regardless of the species.

To address these uncertainties, we carefully examined the immature inflorescences of the type specimen of *Osmoxylon truncatum* in the Kyushu University herbarium (FU) and collected immature inflorescences of the other species recognized here for comparison. Most convincing in our assessment were recent collections of *Osmoxylon oliveri*. Careful examination of immature inflorescences from 17 different trees of *Osmoxylon oliveri* revealed variation in the size and length of inflorescence parts, and the measurements of material ascribed to *Osmoxylon truncatum* fit within this range. More importantly, one collection (Costion 3985) matches the general appearance of inflorescences on the type of *Osmoxylon truncatum* (Suppl. material 1).

To pursue this matter further, we traveled to the type locality of *Osmoxylon truncatum*, Aimeliik on the island of Babeldaob. After observing numerous individuals of *Osmoxylon oliveri*, we made three new collections (Costion 3987, 3988, and 3989) of this species (See Suppl. material 1), all of which contained leaves on young sprouting branches with 5-7 lobes. Costion 3987 was a mature tree along the roadside that had been pruned. All its branches were re-emergent with many 7-lobed leaves. Notably, leaves in upper or higher branches were up to 10-lobed. Costion 3988 was a sapling with 7-lobed leaves that was clearly a juvenile growing directly underneath a fully fruiting, mature individual of *Osmoxylon oliveri*. Costion 3989 was notable in that at the base of the trunk, emerging branches contained 5-7-lobed leaves while more mature leaves in the crown of the tree were 11-13-lobed.

Although there are no known mature inflorescence characters for Kanehira’s species *Osmoxylon truncatum*, the immature characters of both the leaves and inflorescences match those of *Osmoxylon oliveri*. Therefore, we treat these two entities as a single species, *Osmoxylon truncatum*, which has nomenclatural priority.

### Geography

The distribution of the genus *Osmoxylon* is particularly curious, suggesting a pattern of East Malesian bird dispersal. The inflorescence morphology also appears to be perfectly suited for bird pollination. Because the fleshy pseudo-fruits mature as the fertile flowers present pollen, we hypothesize that they may act as a lure to attract birds, who then brush against the fertile flowers of the two lateral peduncles (see also Stone 1962). To date, there have been no published accounts reporting observations on pollination or fruit/seed dispersal in *Osmoxylon*. Locals in Palau report that the Micronesian starling, *Alponis opaca orii*, frequently feeds on the fruits, but these observations do not detail effective pollination nor specify whether the feeding is on the sterile baccate pseudo-fruits or the fertile fruits. We suggest that birds are involved in both pollination (enticed by the pseudo-fruits) and seed dispersal (through the fertile fruits), but observations are needed to record nectar feeding and visits to *Osmoxylon* inflorescences to feed on the fruits and pseudo-fruits by birds or other potential pollinators. Our description of two new species from areas of Palau that have been frequented by professional plant collectors over nearly a century attests to how little is still known about this fascinating genus of plants with such a unique floral and fruiting morphology. We hope this study inspires further data collection on other aspects of these poorly known species.

## Supplementary Material

XML Treatment for Osmoxylon leidichii

XML Treatment for Osmoxylon ngardokense

XML Treatment for Osmoxylon truncatum

XML Treatment for Osmoxylon pachyphyllum

## Supplementary materials

Supplementary material 1 Supporting evidence for the treatment of *Osmoxylon oliveri* and *Osmoxylon truncatum* as one species Data type: species images Explanation note: Photos of flowers taken under microscope. Photos of leaves taken in the field at the type locality of *Osmoxylon truncatum*. This dataset is made available under the Open Database License (http://opendatacommons.org/licenses/odbl/1.0/). The Open Database License (ODbL) is a license agreement intended to allow users to freely share, modify, and use this Dataset while maintaining this same freedom for others, provided that the original source and author(s) are credited.Craig M. Costion, Gregory M. Plunkett
