# Supplementary material for: A revision of the genus Osmoxylon (Araliaceae) in Palau, including two new species
Source: PhytoKeys. 2016 Jan 12;(58):49–64. doi: 10.3897/phytokeys.58.5292 (PMC4743014; doi:10.3897/phytokeys.58.5292)
Supplement: Supplementary material 1 — Supporting evidence for the treatment of Osmoxylon oliveri and Osmoxylon truncatum as one species [file phytokeys-058-049-s001.docx]

**Supporting Information**

**
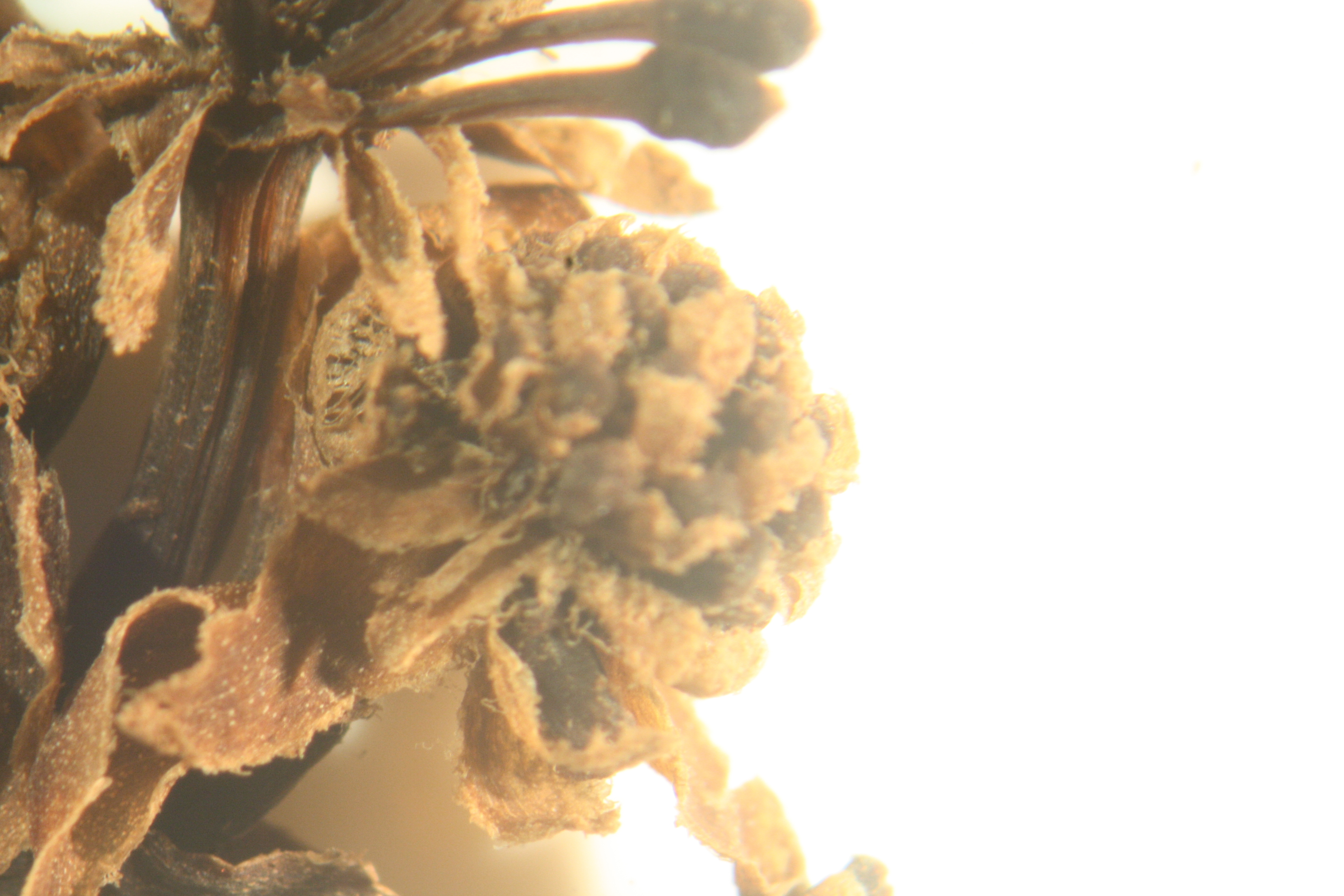

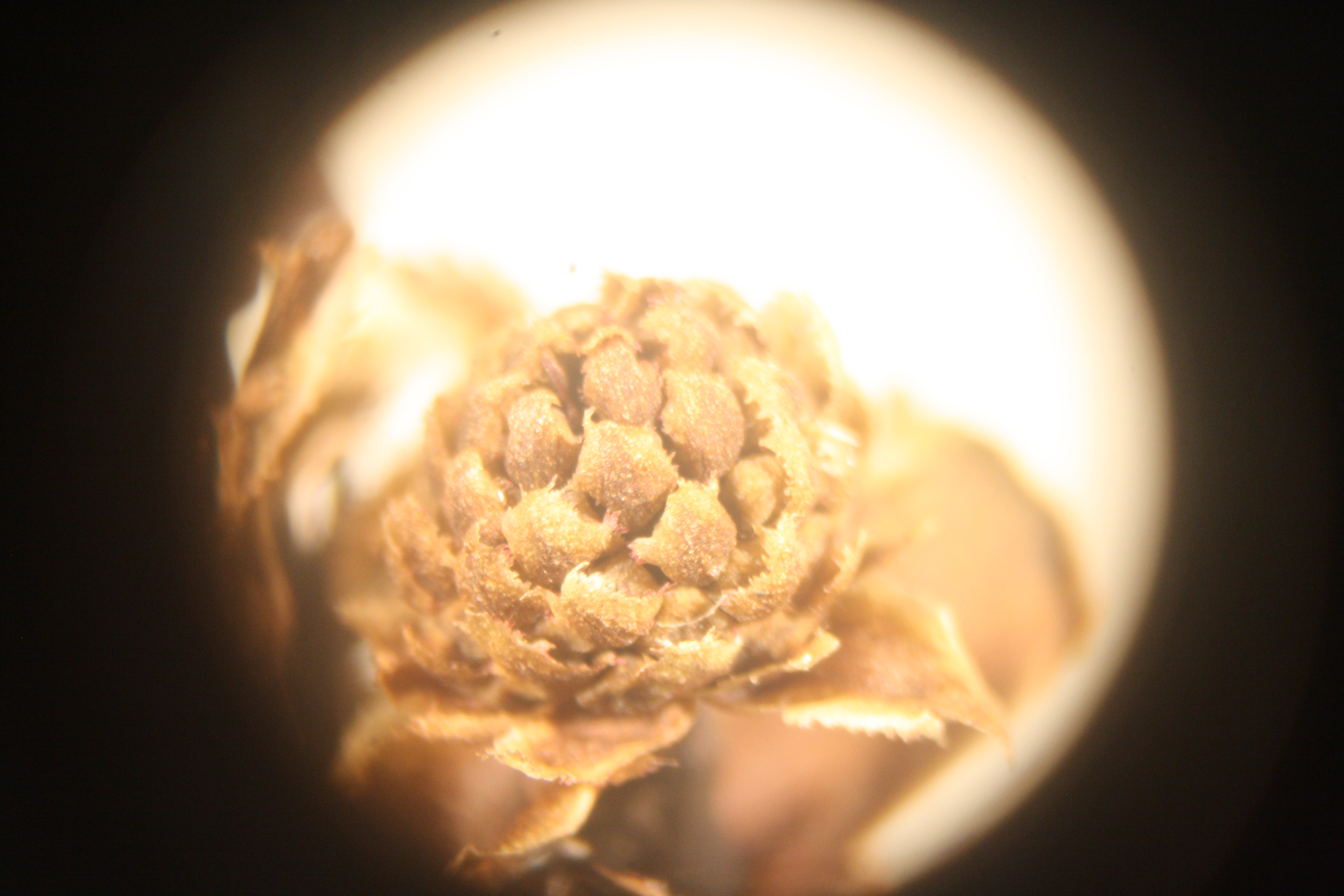
**

**Figure S1:** Immature flower of *O. oliveri* (left) and type specimen of *O. truncatum* (right)


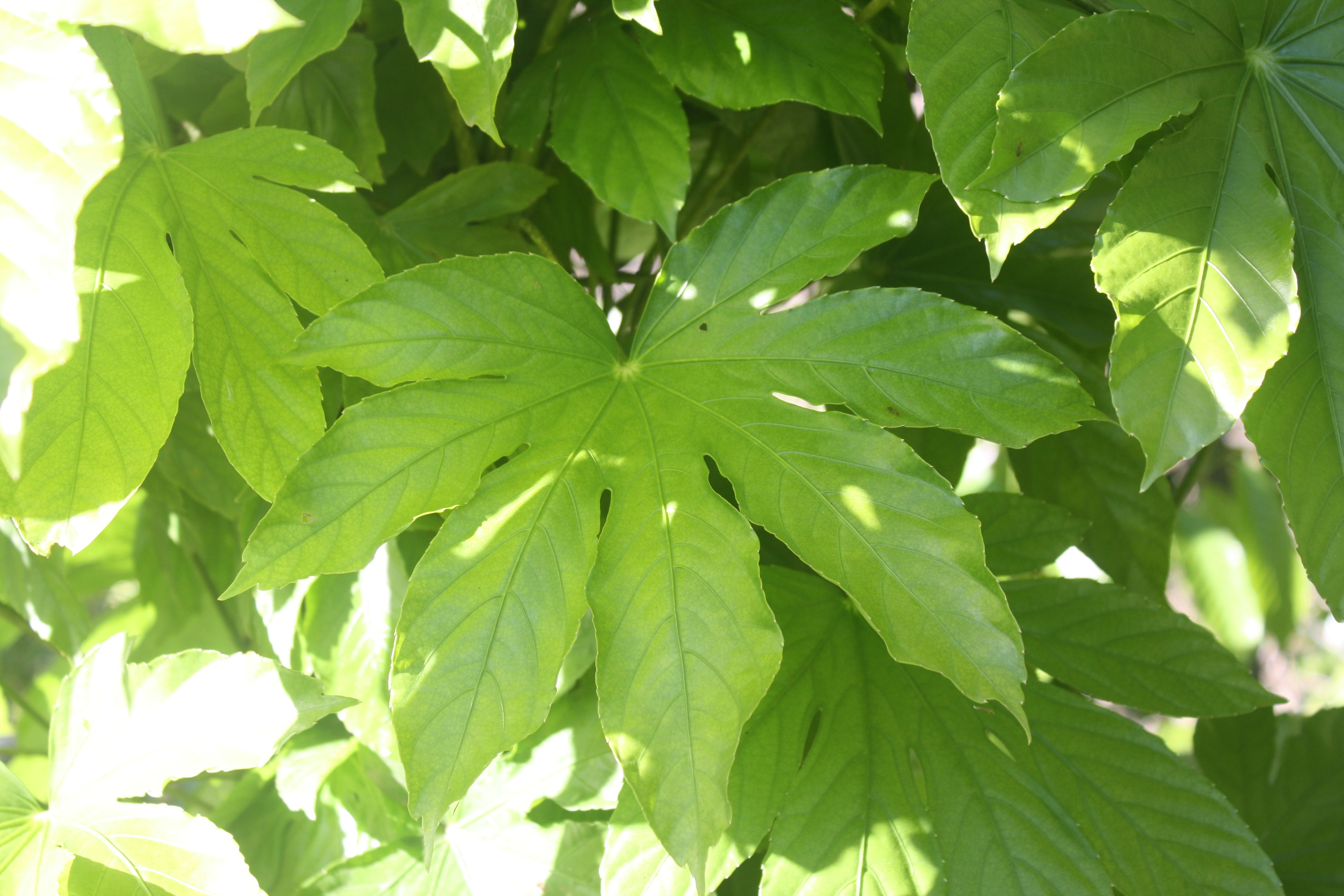


**Figure S2:** Costion 3987, *Osmoxylon oliveri* tree with re-emergent shoots having 7-lobed leaves


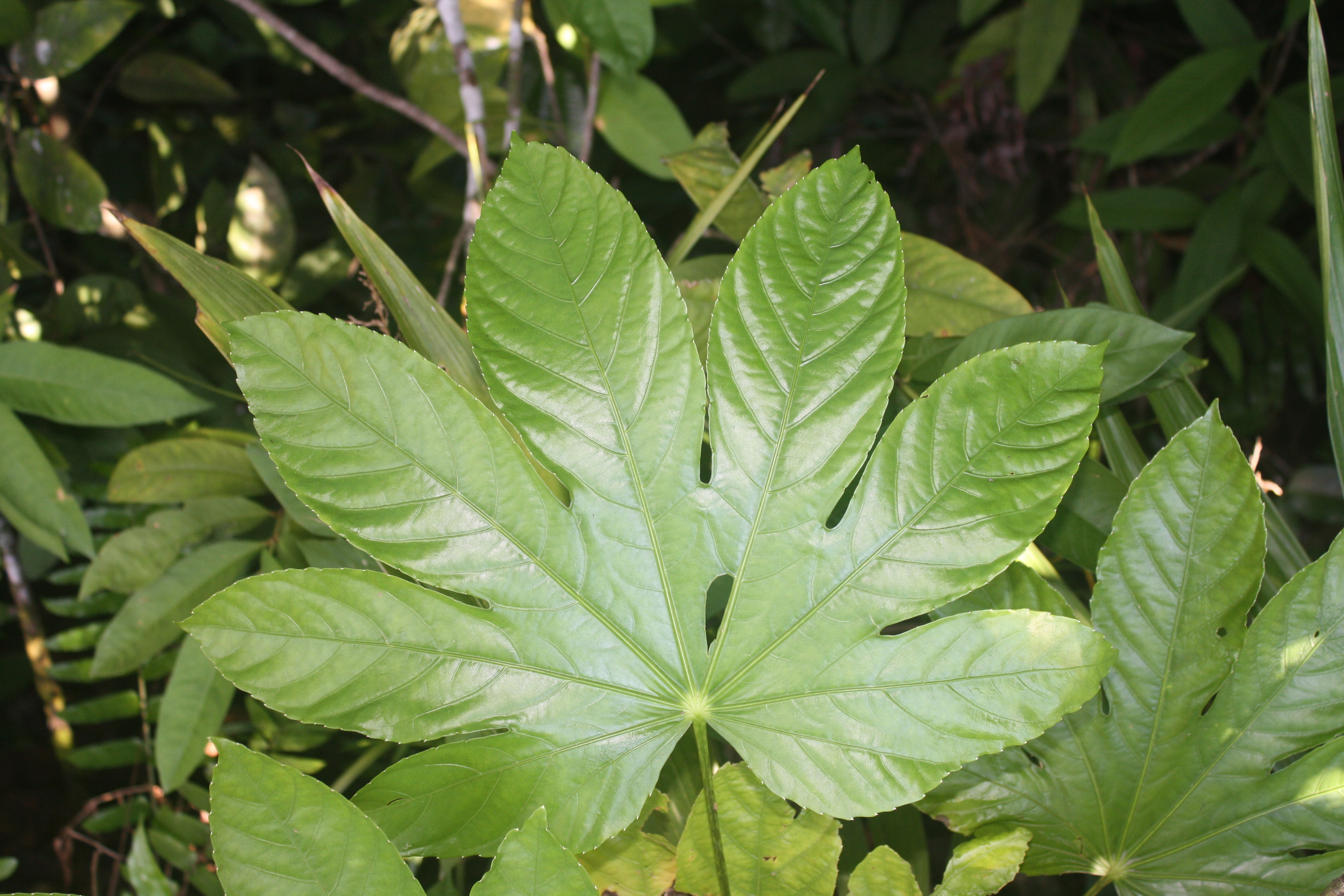


**Figure S3:** Costion 3988, *Osmoxylon oliveri* sapling with 7-lobed, truncate leaves
